# Long-Term Physical Health Outcomes of Resettled Refugee Populations in the United States: A Scoping Review

**DOI:** 10.1007/s10903-021-01146-2

**Published:** 2021-01-30

**Authors:** Gayathri S. Kumar, Jenna A. Beeler, Emma E. Seagle, Emily S. Jentes

**Affiliations:** Immigrant, Refugee, and Migrant Health Branch, Division of Global Migration and Quarantine, National Center for Emerging and Zoonotic Diseases, Centers for Disease Control and Prevention, 1600 Clifton Road, Atlanta, GA 30333 USA

**Keywords:** Refugees, Chronic disease, Non-communicable disease, Physical health

## Abstract

Several studies describe the health of recently resettled refugee populations in the US beyond the first 8 months after arrival. This review summarizes the results of these studies. Scientific articles from five databases published from January 2008 to March 2019 were reviewed. Articles were included if study subjects included any of the top five US resettlement populations during 2008–2018 and if data described long-term physical health outcomes beyond the first 8 months after arrival in the US. Thirty-three studies met the inclusion criteria (1.5%). Refugee adults had higher odds of having a chronic disease compared with non-refugee immigrant adults, and an increased risk for diabetes compared with US-born controls. The most commonly reported chronic diseases among Iraqi, Somali, and Bhutanese refugee adults included diabetes and hypertension. Clinicians should consider screening and evaluating for chronic conditions in the early resettlement period. Further evaluations can build a more comprehensive, long-term health profile of resettled refugees to inform public health practice.

## Introduction

Nearly 750,000 refugees resettled in the US between 2008 and 2019 [1]. Refugees are individuals who are unable to return to their home countries due to a well-founded fear of persecution on account of race, religion, nationality, membership in a particular social group, or political opinion [2]. Before US resettlement, refugee populations often lack access to health services and may have spent years without routine and preventive health care [3], increasing the risk of both communicable and noncommunicable diseases. All US-bound refugees undergo a required health assessment, usually 3 to 6 months before departure for the US [4]. The main purpose of this health assessment is to identify and treat inadmissible medical conditions of public health significance defined by US regulations, rather than a comprehensive medical examination.

After US arrival, refugees are eligible for resettlement and health care benefits [5]. The health benefits for refugees include short-term health insurance in the US for up to 8 months, and a Centers for Disease Control and Prevention (CDC)-recommended domestic medical examination within 90 days of arrival [5]. The CDC *Guidance for the US Domestic Medical Examination for Newly Arrived Refugees* provides guidance to clinicians who conduct the domestic medical examination for refugees [6]. The domestic medical examination includes a thorough review of overseas medical examination forms; a comprehensive medical history; a physical examination; screenings for communicable health conditions; identification of other conditions that may adversely affect resettlement or require further care, such as mental health conditions; preventive health interventions, such as immunizations; and linkage to follow-up primary or specialty care [6]. The health status of refugees shortly after arrival has been well characterized.

After the domestic medical examination and 8 months of short-term health insurance, refugee access to healthcare and insurance varies widely. One study using data from the 2003 New Immigrant Survey documented that up to 50% of resettled refugees were uninsured several years after resettlement [7]. A more recent study reported that 40% of newly arrived refugees resettled to states without Medicaid expansion, reducing the likelihood of Medicaid access beyond the first eight months of arrival in these states (if they remained in that state) [8]. Refugees who do not have access to health insurance or who are underinsured, may seek care at low-cost or free clinics, or acute care settings, such as emergency departments. Since refugees may seek care in diverse health care settings beyond the first 8 months, it is important for clinicians serving refugees in these settings to understand health conditions most commonly encountered in refugees.

Therefore, we conducted a scoping review of the literature to summarize the long-term physical health outcomes of refugee populations beyond the first 8 months of arrival to the US. Given the changing demographic landscape of arriving refugee populations, we limited the review to the top five recently resettled refugee populations over the last 10 years (2008–2018). These populations include Burmese, Iraqi, Bhutanese, Somali, and Congolese refugees.

## Methods

### Identification of Relevant Articles

We reviewed scientific articles published in English from January 2008 to March 2019 using five electronic databases: Medline, PsycInfo, CINAHL, Scopus, and Sociological Abstracts (ProQuest). Research and non-research articles were both included. Search terms within the article title or abstract were kept broad to increase the likelihood of identifying relevant publications and included: (a) “refugee” or “migrant population” or “asylum seeker” *and* (b) *“*United States” or any of the 50 US states. Given that the current article summarizes results from previously published articles, the scoping review was determined to not require formal Institutional Review Board review.

**Fig. 1:**
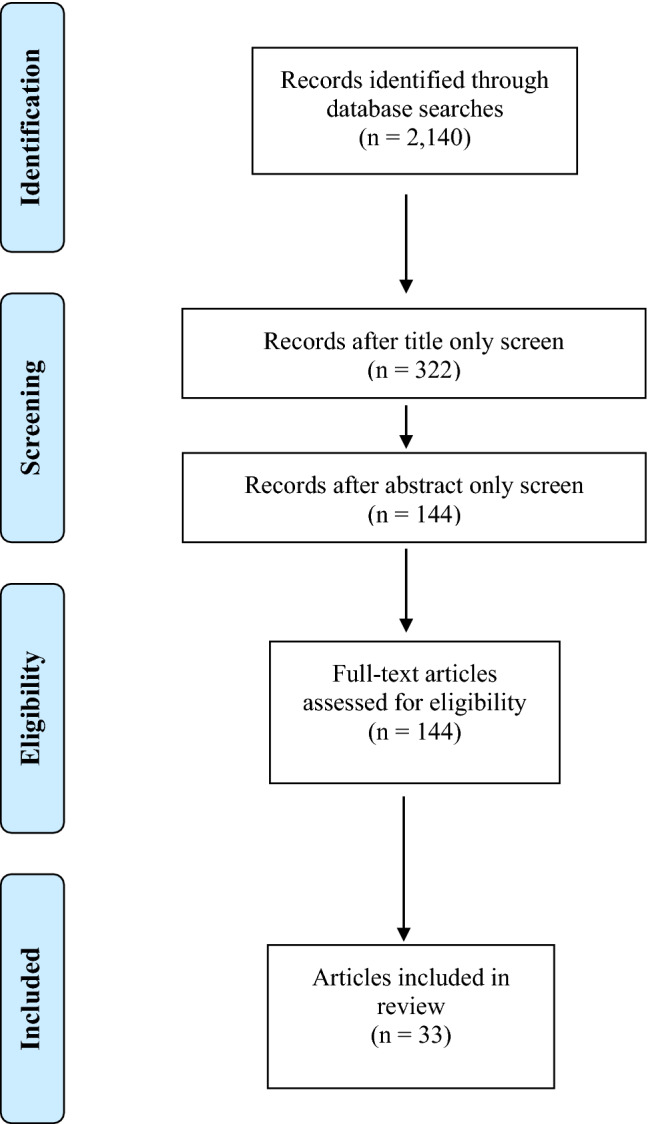
Flow of identification, screening, and review of articles during the literature review

The primary article inclusion criteria were refugees from one of the top five resettled refugee populations (Burmese, Iraqi, Bhutanese, Somali, or Congolese) in the US over the last ten years (2008–2018) or refugees from an unspecified population, and data describing physical health outcomes beyond the first eight months of arrival in the US. Thus, article titles were first reviewed to see if the article met the inclusion criteria (Fig. 1) and then abstracts were reviewed. Finally, selected articles were thoroughly reviewed. If it was unclear whether an article met the inclusion criteria based on the title and abstract screen, it was included in the full review.

Articles were excluded if the article:

- did not mention refugees
- did not assess physical health outcomes (e.g., mental health/preventive health outcomes and assessment of knowledge and attitudes towards care)
- assessed impact of interventions with refugee populations
- included only data from refugees who were in the US for less than 8 months
- included only data on refugees who resettled in a country other than the US
- specifically focused on refugee populations that did not include at least one of the top 5 resettled refugee populations in the US in the last 10 years (e.g., Cambodian)
- did not include primary data (e.g., literature reviews, meta-analyses, theoretical or perspective commentaries)
- abstracts of proceedings or dissertations were also excluded.

### Measures

A data abstraction tool was developed to collect relevant information from each article. Information collected included article title, publication year, author names, investigation study period, refugee population(s) included in the article, sample size of studied population, location that the investigation took place, study design, primary study measures, comparison group (if included), relevant sociodemographic characteristics of the studied population, quantitative data describing primary physical health outcomes, and any study limitations. Physical health outcomes were defined as any outcome that impacts the physical body, unrelated to mental and social well-being. Examples of primary physical health outcomes include communicable diseases such as Hepatitis B and C, and non-communicable conditions such as diabetes, heart disease, vitamin D deficiency, and elevated blood lead levels (EBLL).

### Data Collection and Synthesis

One author reviewed the article titles, abstracts, and, if needed, full article to see if articles met the inclusion criteria. After article selection following this initial screening, 3 authors completed the full article review, which included data abstraction. At least two authors completed data abstraction for each article, with one author completing data abstraction of all articles. If there were any discrepancies in the information collected from the article during data abstraction or disagreement as to whether an article met criteria for inclusion, the two authors discussed until consensus was reached.

Following data abstraction, primary quantitative findings from articles were summarized and included in the current report. Data were also reported if similar outcomes were reported across articles or if a comparison population was included in the article (i.e., data from the comparison population was also included in the article). Given that outcomes measures (e.g., prevalence vs. incidence or odds ratios vs. hazard ratios) were reported differently across most articles, we directly report the outcomes from the article and did not conduct additional analyses with the reported data.

## Results

### Article Selection

Initial search results from all five included databases yielded 2140 articles, 33 of which (1.5%) met the inclusion criteria and were included in the final review and data abstraction.

### Characteristics of Included Articles

Among the 33 articles included in this review, 16 articles (48.5%) included all refugee populations (which may include the top 5 resettled refugee populations that met the inclusion criteria), 5 focused on Bhutanese refugees (15.2%), 4 on Somali refugees (12.1%), 4 on Iraqi refugees (12.1%), 3 on African refugees (9.0%) and 1 on Burmese refugees (0.3%). No articles described physical health outcomes specifically among the Congolese refugee population, although articles that included all refugee populations may have included Congolese refugees. Of the 33 included articles, 17 (51.5%) were retrospective chart reviews, 13 (39.4%) used a cross-sectional study design, and 3 (9.0%) used a prospective study design. Investigations took place across 16 states with the majority taking place in Minnesota (seven articles, 21%); other states included Michigan (4), New York (3), Rhode Island (3), Washington (3), Massachusetts (2), Ohio (2), Pennsylvania (2), Texas (2), California (1), Georgia (1), Idaho (1), Illinois (1), Indiana (1), Kentucky (1), and Oregon (1). Two investigations took place across multiple states [9, 10], and 2 investigations analyzed data from the New Immigrant Survey (fielded in 2003), which used a nationally representative sample of refugees and immigrants [7, 11]. Most investigations took place in health care settings (23, 67.6%). Nine (28, 1.3%) articles compared refugee populations to other populations such as non-refugee immigrants, US-born patients, and patients who did not use interpreter services.

The following sections summarize the main findings for all resettled refugee populations and the 4 leading resettled refugee populations by volume during 2008–2018 (Bhutanese, Somali, Iraq, Burmese).

#### All Refugees

Among the 16 publications looking at physical health outcomes among all resettled refugee populations, 9 articles specific refugee adults as the target population [7, 11–18], and 7 articles specified refugee children [9, 19–24]. Most articles did not specify countries of origin, but rather grouped refugee country of origin into geographic regions (e.g., Southeast Asia and Africa). Three articles focused on African refugees. Therefore, results were summarized separately for these articles [25–27].

##### Adults

The majority of the articles on refugee adult populations reported chronic disease outcomes. Refugee adults had a higher prevalence (21–25%) and up to 2 times the odds of having any chronic condition compared to non-refugee immigrant adults (13–16%) [7, 11]. Non-refugee immigrants in these studies denoted individuals who received legal permanent residency (LPR) in the United States, but not via refugee visa. Individuals were categorized based on their pre-LPR visa category, which included temporary students, workers or visitors; undocumented immigrants; or immigrant spouses, fiancés, or children of US citizens or LPRs. Compared to non-refugee immigrant populations in these studies, a greater proportion of refugee populations were from Europe and Central Asia (34% vs. 11% for non-refugee immigrant adults) and Sub-Saharan Africa (12% vs. 4%), were poor (24% vs. 18%), and had limited English proficiency (49% vs. 39%) [7]. Refugees were more likely to be in the US for a shorter period of time than non-refugee immigrants (mean: 78 months vs. 110 months) [7]. For specific conditions, refugees were more likely than non-refugee immigrants to self-report having conditions such as arthritis (7% vs. 3%; OR = 1.7, 95% CI 1.1–2.6) and heart disease (3% vs. 1%; OR = 2.5, 95% CI 1.3–4.7) [7].

Four articles reported body-mass-index (BMI), diabetes, and hypertension outcomes [12, 15, 16, 18]. In one study, refugee adults had an overall increase in BMI of 0.72 kg/m^2^ over 3 months from baseline measurement [12]. However, adults from the Middle East had a higher average baseline BMI of 27.5 kg/m^2^ compared to adults from Africa (21.0 kg/m^2^) and Southeast Asia (21.8 kg/m^2^). Compared to non-refugee immigrant adults, refugees had a greater prevalence of diabetes (5–6% vs. 3–4% for non-refugee immigrant adults) [7, 11] and hypertension (12–14% vs. 6–9%) [7, 11]. Compared with age- and gender-matched US-born controls, refugees had an increased risk for diabetes (hazard ratio: 2.1; 95% CI 1.3–3.3) [18], with a diabetes incidence rate of 1.94 per 100 person-years (vs. 1.22 per 100 person-years for US-born controls). Of note, compared to US-born controls in this study, a greater proportion of refugees had less than a high school diploma (42% vs. 11% for US-born controls) and had a lower median household income ($40,099 vs. $48,328) [18]. Among refugees, there was an estimated 12% odds of diabetes (OR: 1.12; 95% CI 1.03–1.2) and 7% odds of hypertension (OR 1.07; 95% CI 1.0–1.1) for each year post-resettlement to the US [16].

Vitamin D deficiency was more prevalent among refugee and immigrant populations (60%) compared to the US-born population (35%); refugees and immigrants also had a higher odds of vitamin D deficiency (OR: 3.0; 95% CI 2.1–4.4) and severe vitamin D deficiency (OR: 2.2; 95% CI 1.2–4.1) [14]. Among refugee adults previously diagnosed with human immuno-deficiency virus (HIV), the majority (59%) had Stage I (asymptomatic) disease [13]. In another analysis, about 8% of refugee adults were reported to have chronic hepatitis B virus (HBV) infection [17].

##### Children

Most articles on resettled refugee children reported changes in anthropometric measurements over time. Overweight and obesity prevalence increased post-resettlement, with prevalence at baseline (shortly after arrival) ranging from 9–17% and increasing to 21–35% several years post-resettlement [9, 19]. For overweight, baseline estimates ranged from 14–18% [19, 20] and increased to 23–31% [19, 20]. For obesity, baseline estimates ranged from 0–7% [9, 19, 20] and increased to 13–18% [9, 19, 20]. In one study, the majority of underweight children at arrival reached normal weight after 1 year (57%) [24], although this was more pronounced in older children [24]. In another study, refugees had a steeper increase in their BMI z-score over 12 months compared with non-refugees (coefficient: 0.18 vs 0.03; p < 0.001) who were matched to the refugee cohort by age, sex, and year of care initiation [9]. In this same study, at baseline, a greater proportion of refugees had wasting (5% vs. 2% for non-refugees) and stunting (9% vs 2%) and had a lower BMI z-score (mean: − 0.3 vs. 0.7) compared to non-refugees [9].

Few articles reported malnutrition outcomes among refugee children [9, 21]. In one study, the prevalence of acute malnutrition and chronic malnutrition were not significantly different from date of first measurement (acute malnutrition: 5%; chronic malnutrition: 9%) to the date of last measurement [9]. Another article revealed that average height-for-age (HFA) z scores increased from time of initial assessment over the next five years (baseline range: − 1.36 to − 1.13; 5 years: − 0.79) [21].

Other non-communicable diseases reported include vitamin D deficiency (87%) [21], anemia (25%), [21, 23] and lead poisoning (elevated blood lead levels [EBLL]; 6–11%) [21, 22]. Communicable diseases reported among refugee children include tuberculosis (positive purified protein derivative (PPD) test prevalence: 15–23%) [21, 23], chronic hepatitis B infection (4%) [21], and intestinal parasite infection (12–22% with *Giardia*) [21].

#### African Refugees

Three articles described physical health outcomes among resettled African refugee populations [25–27], with 1 article reporting results among adults [25] and 2 articles reporting results among adults and children [26, 27]. Among adults, in one study, the most common non-communicable diseases reported include elevated blood pressure (18%), elevated cholesterol (18%), diabetes (17%), and overweight (8%) [27]. Among children, the most commonly reported conditions include asthma (4%) and overweight (3%) [27]. Compared with non-refugee adults who were matched to African refugee adults by age, gender, race, and year of visit, African refugee adults had a significantly lower prevalence of obesity (27 vs. 41%; p = 0.02) and hypertension (19 vs. 35%; p = 0.005) at 5 years following the initial measurement [25]. Using paired refugee data, there was a significant increase from initial measurement to year 5 for diabetes (1.0 vs. 10.8%), hyperlipidemia (3.9 vs. 10.8%), hypertension (16.7 vs. 21.6%), and obesity (12.8 vs. 27.5%) [25].

About 8% of resettled African refugees were positive for hepatitis B surface antigen [26]. Of those with complete serological data, 37% had evidence of past infection, 29% were immune, and 26% were susceptible to the virus [26].

##### Bhutanese

All 5 articles of health outcomes among resettled Bhutanese refugees reported estimates of the prevalence of non-communicable diseases [28–32]. Four articles reported outcomes among adults. In one study, approximately 59% had at least 1 chronic disease [31]. The most commonly reported chronic diseases among Bhutanese refugee adults include arthritis (22%) [29], overweight/obesity (4–52%) [28–31], hypertension (19–27%) [28–30], diabetes (6–14%) [28–30], and asthma (6–7%) [29, 30], with higher estimates reported from studies of medical chart review data (vs. self-reported data) [28, 30]. Vitamin B_12_ deficiency estimates among Bhutanese refugees ranged from 12–32% [28, 32].

##### Somali

Four articles reported health outcomes among adult Somali refugees resettled to the US. Three articles reported prevalence estimates of chronic diseases [33–35], and 1 reported oral health outcomes [36]. Commonly reported chronic diseases among the Somali population include obesity (35–41%) [33, 34] and overweight (33–35%) [33, 34], hypertension (17–32%) [33, 34], diabetes (12–24%) [33, 34] and dyslipidemia (18%) [34]. In one study, compared with age-quartile matched non-Somali patients, Somali patients had a greater prevalence and/or odds of prediabetes (21% vs 17% for non-Somali; OR: 1.6, 95% CI 1.2–2.1) [34], diabetes (12% vs 5%; OR: 2.8, 95% CI 1.8–4.4) [34], overweight (33% vs. 30%; p = 0.047) [34] and obesity (35% vs 32%) [34]. In this study, fewer Somali patients had higher level education (some college: 27% vs. 78% for non-Somali patients) and were employed (36% vs. 66%) compared to non-Somali patients [34]. Periodontal disease was noted in 6.5% of the Somali refugee population; participants had an average of 5.5 decayed, missing and filled teeth [36].

##### Iraqi

All four articles reporting outcomes among resettled Iraqi refugee populations described chronic disease outcomes among adults, with three using self-reported survey data [10, 37, 38]. Approximately 60% of Iraqi adults reported having at least 1 chronic condition, while 37% reported having at least 2 chronic conditions [38]. The most commonly reported health conditions include fatigue (63%) [38], elevated cholesterol (34%) [10], hypertension (13–35%) [10, 37, 38], musculoskeletal system problems (29%) [38], and diabetes (6–16%) [10, 37]. Overweight and obesity prevalence at 1 year following resettlement were approximately 38% and 22%, respectively [37]. In one study, Iraqi women had a significantly higher increase in BMI than men at 2 years following resettlement (1.4 ± 0.2 kg/m^2^ and 0.4 ± 0.2 kg/m^2^, respectively) [39].

##### Burmese

Only 1 article described physical health outcomes in the resettled Burmese refugee population [40] and reported the prevalence of EBLLs among Burmese refugee children in Indiana [40]. Approximately 37% of children had blood lead levels (BLL) > 5 µg/dL and 7% had BLLs > 10 µg/dL. Children with daily use of *thanakha* (a cosmetic widely used by Burmese people) had a higher geometric mean BLL of 9.6 µg/dL compared to those with no reported or infrequent use.

## Discussion

Of the articles included in our review of physical health outcomes beyond the first 8 months of resettlement, we found that most studies among refugee populations reported non-communicable disease health outcomes, specifically chronic disease outcomes. In a limited number of studies with controls, refugee adults were reported to have higher prevalence and/or odds of having chronic disease conditions, including diabetes and hypertension, compared with non-refugee immigrant adults, and an increased risk for diabetes and Vitamin D deficiency compared with the US population. Among Iraqi, Somali, and Bhutanese refugee adults, commonly reported chronic diseases included diabetes, hypertension, and obesity. Among all refugee children, prevalence of overweight and obesity increased over time. We have organized the discussion for our review as follows: (1) summary of potential contributing factors for chronic diseases among refugee populations; (2) clinical recommendations for screening and management of chronic diseases among resettled refugee populations; (3) preventive health recommendations for chronic disease prevention and control among resettled refugee populations and (4) limitations of our review.

Chronic diseases were the most commonly reported physical health outcomes among resettled refugee populations in the US. However, the studies primarily reported on chronic disease conditions, with few reporting communicable disease conditions. Furthermore, several studies noted that refugee adults have higher prevalence and/or odds of chronic diseases compared with other populations. Several factors may influence risk or progression of chronic diseases among refugee populations including individual-level factors such as reduced consumption of healthy foods (e.g., reduced fruit and vegetable intake) [29, 41–43], reduced physical activity [29, 41, 42, 44, 45], limited health literacy [46], varied perceptions and beliefs on health and health care [47–49], limited English proficiency and subsequent challenges in communication [49–51], and reduced access to health care [7, 48, 50, 51]. While there may be multiple reasons for decreased healthy eating among resettled refugee populations in the US (relative to the US general population), acculturation has been identified as an important contributor. Acculturation has been associated with increased risk of obesity and cardiovascular disease, mediated in part by suboptimal dietary behaviors (such as increased fast food intake) [44, 52, 53]. Additionally, up to 50% of refugees may be uninsured > 8 months after arrival (with 47% of refugees with chronic conditions being uninsured) [7], limiting their ability to access health care services for monitoring and control of health conditions [7, 29, 42]. Community-level factors such as decreased availability of healthy foods [42, 54], spaces to be active [39], health care facilities (especially in resettlement communities) and community transportation options to health facilities [48, 51] may also contribute to increased risk of development or progression of chronic diseases among resettled refugee populations.

Clinical and preventive health guidance for long-term care of resettled refugee populations in the US is limited. The CDC *Guidance for the U.S. Domestic Medical Examination for Newly Arriving Refugees* provides guidance to state public health departments and clinicians conducting the domestic medical examination of newly arrived refugees within the first 90 days of arrival [6]. In addition, other state health departments have adapted the CDC guidance or have developed clinical tools, such as the online interactive tool called CareRef [55], to assist clinicians in conducting the domestic medical examination. Based on the summarized findings from this scoping review, clinicians should also consider screening and evaluating for a broader range of non-communicable diseases in the early resettlement period (≤ 8 months of arrival), in addition to those recommended by the CDC guidance. Ability to screen for these conditions earlier may be dictated by availability of and accessibility to long-term primary care, including existing health insurance policies available for these populations in a state. If possible, clinicians should consider screening for non-communicable diseases identified through this review, which include diabetes among those with risk factors and dyslipidemia among adults. Prompt referral and management of other chronic diseases such as hypertension and overweight/obesity are also important. Beyond the first 8 months of resettlement, clinicians who treat resettled refugees in health care settings can refer to US clinical guidelines (e.g., United States Preventive Services Task Force and American Diabetes Association) for screening and management of several non-communicable diseases based on age and risk factors, including prediabetes and diabetes, dyslipidemia, and hypertension [56–60], although variations across these guidelines exist. Given the higher risk of some non-communicable diseases among resettled refugee populations and inconsistent access to health care, community-level interventions to improve screening of chronic diseases such as diabetes and dyslipidemia should also be considered.

In addition to screening for and managing certain non-communicable diseases, behavioral risk factor screening and counseling for those with cardiovascular disease risk factors (e.g., diet, physical activity, and smoking behaviors) [61], and referral to culturally appropriate chronic disease prevention and management programs can be initiated at the domestic medical examination. Primary care providers and other health care professionals should continue to reemphasize healthy lifestyle practices and refer to available programs. For instance, refugees with prediabetes or chronic diseases could be referred to available evidence-based chronic disease prevention and management programs such as the National Diabetes Prevention Program and Chronic Disease Self-Management Programs [62, 63] which would need to be culturally and linguistically adapted for specific refugee populations. Home-based educational interventions among immigrant populations have been shown to improve dietary quality and physical activity over 24 months [64].

While several studies have described barriers to health and health care among resettled refugee populations, interventions to reduce such barriers are needed. Resettlement agencies play a crucial role in ameliorating challenges with health care access in the early resettlement period. However, after refugees no longer receive resettlement agency support (which could vary from a few months to up to several years depending upon the agencies’ programs), they are at a greater risk of becoming uninsured or underinsured [50]. Community-based interventions such as community health workers can facilitate removing some of these barriers, such as linking refugees with health insurance or free or low-cost health care (if health insurance options are unavailable), and have the potential to empower refugees to assume charge of their health and seek care when appropriate [50].

This review has several limitations. First, although extensive, our literature search may have missed some relevant publications. For example, publications only referring to immigrant or foreign-born populations but that do not explicitly mention refugees, were excluded. It is possible that some of these studies included refugees. Second, the inclusion criteria may have limited the scope and depth of this review. Multiple studies describe preventive health, health access, and other outcomes among resettled refugee populations but were not included; however, summaries of these outcomes could be described in future reviews. Third, included studies describing foreign-born populations that include refugees may also include other populations such as Special Immigrant Visa holders, asylees, and undocumented migrants. Health profiles of refugee and non-refugee populations even from the same country of origin can vary significantly due to differences in socio-economic status, ethnic background, migration patterns, and education. Fourth, most studies included in this review were retrospective chart reviews from health care settings. Retrospective analyses often provide a snapshot of health, although some describe changes in health metrics over an extended time period. Further, refugees who seek care in health care settings are likely more medically complex given that they are presenting for either acute health concerns or management of chronic health concerns; therefore, the prevalence of disease in their respective population may be overestimated. Fifth, while several studies included comparison populations (see Tables 1 and 2), most did not. Such information could help identify differences in health outcomes between resettled refugees and other populations. Sixth, data for pediatric patients were limited, and most studies focused on changes in anthropometric measurements over time. Seventh, this review only provides a summary of major pertinent findings, not necessarily all study results. Eighth, most studies report that there is limited ability to follow refugees long-term. For example, refugees may choose to change clinical providers (resulting in missing records). Ninth, there is a possibility of reporting bias in that most articles primarily report chronic disease outcomes given that these diseases are typically screened and monitored for in the US general population.

**Table 1:** Summary of primary findings of review of longer-term physical health outcomes among resettled refugee adults in the United States^a^

Refugee population	No. of studies	Sample size	Summary of primary findings	Comparison group(s)
*All refugees*				
Chronic conditions	2	480–549 (refugees) 3715–7654 (non-refugee immigrants)	Higher prevalence of having a chronic condition compared to non-refugee immigrants (21–25% vs. 13–16%) [7, 11] Greater odds of having a chronic condition compared to non-refugee immigrants (OR: 1.9 [95%CI 1.4–2.5]) [7, 11]	Non-refugee immigrants [7, 11]^b^
Diabetes	4	478–3174 (refugees) 3715–7654 (non-refugee immigrants)	Prevalence of diabetes ranged from 5–6% [7, 11] Increased risk of diabetes compared to US-born controls (HR: 2.1; 95% CI 1.3–3.3) [18] Estimated 12% odds of developing diabetes for each year post-resettlement to the US (OR: 1.12; 95% CI 1.0–1.2) [16]	Non-refugee immigrants [7, 11]^b^
Hypertension	3	480–559 (refugees) 3715–7654 (non-refugee immigrants)	Prevalence of hypertension ranged from 12–14% [7, 11] Estimated 7% odds of developing hypertension for each year post-resettlement to the US (OR 1.07; 95% CI 1.0–1.1) [16]	Non-refugee immigrants [7, 11]^b^
Vitamin D deficiency	1	1378	Higher prevalence of Vitamin D deficiency compared to US-born population (60% vs. 35%) [14] Greater odds of Vitamin D deficiency (OR: 3.0; 95% CI 2.1–4.4) [14]	US-born population [14]
*African refugees*				
Diabetes	2	220–260	Prevalence of diabetes ranged from 9–17% [25, 27]	
Dyslipidemia	2	133–260 (refugees) 133 (non-refugees)	Prevalence of elevated cholesterol ranged from 8–18% [25, 27] Lower prevalence of hyperlipidemia compared to non-refugees (8% vs. 12%) [25]	Non-refugees [25]
Hypertension	2	133 (refugees) 133 (non-refugees)	Lower prevalence of hypertension compared to non-refugees (19% vs. 35%) [25]	Non-refugees [25]
Obesity/overweight	1	133 (refugees) 133 (non-refugees)	Prevalence of obesity was 27% [25] Lower prevalence of obesity compared to non-refugees (27% vs. 41%) [25]	Non-refugees [25]
*Bhutanese*				
Diabetes	3	66–120	Prevalence of diabetes ranged from 6–14% [28–30]	–
Hypertension	3	66–120	Prevalence of hypertension ranged from 19–27% [28–30]	–
Overweight/obesity	4	66–120	Prevalence of overweight/obesity ranged from 4–52% [28–31]	–
Vitamin B_12_ deficiency	2	66–141	Prevalence of Vitamin B deficiency ranged from 12–32% [28, 32]	–
*Somali*				
Prediabetes/diabetes	2	72–1007 (Somali) 1010 (non-Somali)	Higher prevalence of prediabetes compared to non-Somali patients (21% vs. 17%) [34] Greater odds of prediabetes (OR: 1.6; 95% CI 1.2–2.1) [34] Prevalence of diabetes ranged from 12–24% [33, 34] Greater odds of diabetes (OR: 2.8; 95% CI 1.8–4.4) [34]	Non-Somali patients [34]
Hypertension	2	72–1007 (Somali)	Prevalence of hypertension ranged from 17–32% [33, 34]	
Overweight/obesity	2	72–1007 (Somali) 1010 (non-Somali)	Prevalence of obesity ranged from 35–41% [33, 34]	Non-Somali patients [34]
*Iraqi*				
Chronic conditions	2	366	Prevalence of ≥ 1 chronic condition was 60% [37]	–
Diabetes	2	290–366	Prevalence of diabetes ranged from 6–16% [37, 38]	–
Hypertension	3	290–366	Prevalence of hypertension ranged from 13–35% [37, 38, 40]	–
Overweight/obesity	2	290	Prevalence of overweight and obesity was 38% [38] Prevalence of obesity was 22% [38] Greater increase in BMI among females (1.4 kg/m^2^) compared to males (0.4 kg/m^2^) [40]	–

*OR* odds ratio, *CI* confidence interval, *BMI* body-mass-index (kg/m^2^)

^a^Includes top 5 resettled refugee populations by volume in the United States: Burmese, Iraqi, Bhutanese, Somali, and Congolese. Not all findings from studies are included in the table

^b^Non-refugee immigrants in these studies denote individuals who received legal permanent residency in the United States, but not via refugee visa. Individuals were categorized based on their pre-LPR visa category, which included temporary students, workers or visitors; undocumented immigrants; or immigrant spouses, fiancés, or children of US citizens or LPRs

**Table 2:** Summary of primary findings of review of longer-term physical health outcomes among resettled refugee children in the United States^a^

Refugee population	No. of studies	Sample size	Summary of primary findings
*All refugees*			
Overweight/obesity	4	181–1067	Baseline prevalence of overweight and obesity ranged from 9–17% and increased to 21–35% several years post-resettlement [9, 19, 20]
Acute/chronic malnutrition	2	199–512	Prevalence of acute and chronic malnutrition were not different from time of first measurement (acute malnutrition: 5%; chronic malnutrition: 9%) to time of last measurement [9] Average height-for-age z scores increased from time of initial assessment over the next five years (baseline range: − 1.36 to − 1.13; five years: − 0.79) [21]
Elevated blood lead levels (EBLL)	2	199–1726	Prevalence of EBLL ranged between 6–11% [21, 22]
Vitamin D deficiency	1	199–225	Prevalence of Vitamin D deficiency was 87% [21]
Anemia	2	198–225	Prevalence of anemia was 25% [21, 23]
Tuberculosis	2	198–225	Prevalence of latent tuberculosis was 61% [23] Prevalence of a positive PPD result ranged from 15 to 23% [21]
*Burmese*			
EBLL	1	197	Prevalence of EBLL > 5 mcg/dL was 37% [41] Prevalence of EBLL > 10 mcg/dL was 7% [41]

*EBLL* elevated blood lead level

^a^Includes top 5 resettled refugee populations by volume in the United States: Burmese, Iraqi, Bhutanese, Somali, and Congolese. Not all findings from studies are included in the table

## Conclusions

Chronic disease outcomes were the most commonly reported outcomes among resettled refugees in the studies included in our review. This is not surprising given that chronic diseases are some of the leading contributors of disability and death among the general US population, with six in ten US adults having a chronic disease [65]. Given the increased incidence and prevalence of some chronic diseases among refugees over time, we recommend earlier screening and evaluation of some non-communicable diseases—in addition to those recommended by the CDC guidance—provided that refugees are able to access longer-term primary care. It would also be beneficial if primary care clinicians are trained in refugee healthcare. In order to fully understand the health needs of resettled refugee patients in the US, further analyses are needed. Additional prospective studies that assess longitudinal physical health outcomes among all refugees and specific refugee populations will allow public health practitioners and clinicians to gain a better understanding of health conditions post-resettlement. Mechanisms to track refugee health status over time need to be incorporated into medical record systems. More analyses among refugee children are also needed to determine conditions that may persist or develop as children reach different stages of development. These assessments can build a more comprehensive health profile of resettled refugees to inform public health practice and allow for targeted screening and clinical interventions beyond the initial resettlement period.
